# Rare and novel variants of *PRKN* and *PINK1* genes in Vietnamese patients with early‐onset Parkinson’s disease

**DOI:** 10.1002/mgg3.1463

**Published:** 2020-08-27

**Authors:** Nguyen Dang Ton, Nguyen Duc Thuan, Ma Thi Huyen Thuong, Tran Thi Bich Ngoc, Vu Phuong Nhung, Nguyen Thi Thanh Hoa, Nguyen Hoai Nam, Hoang Thi Dung, Nhu Dinh Son, Nguyen Van Ba, Nguyen Duy Bac, Tran Ngoc Tai, Le Thi Kim Dung, Nguyen Trong Hung, Nguyen Thuy Duong, Nguyen Hai Ha, Nong Van Hai

**Affiliations:** ^1^ Institute of Genome Research Vietnam Academy of Science and Technology Hanoi Vietnam; ^2^ Graduate University of Science and Technology, Vietnam Academy of Science and Technology Hanoi Vietnam; ^3^ 103 Military Hospital, Vietnam Military Medical University Hanoi Vietnam; ^4^ Vietnam Military Medical University Hanoi Vietnam; ^5^ University Medical Center HCMC University of Medicine and Pharmacy at HCMC Ho Chi Minh City Vietnam; ^6^ National Geriatric Hospital Hanoi Vietnam

**Keywords:** early‐onset Parkinson's disease, *PARKIN*, *PINK1*, *Vietnamese*

## Abstract

**Background:**

Early‐onset Parkinson's disease (EOPD) refers to that of patients who have been diagnosed or had onset of motor symptoms before age 50, accounting for 4% of Parkinson's disease patients. The *PRKN* and *PINK1* genes, both involved in a metabolic pathway, are associated with EOPD.

**Methods:**

To identify variants associated with EOPD, coding region of *PARKIN* and *PINK1* genes in 112 patients and 112 healthy individuals were sequenced. Multiplex ligation‐dependent probe amplification kit was used to determine EOPD patients that carried mutations in *PRKN* and *PINK1* genes.

**Results and Conclusion:**

Three rare and three novel mutations in total of 14 variants of *PARKIN* and *PINK1* were detected in the EOPD cohorts. Mutations of *PRKN* and *PINK1* genes were found in five (4.4%) patients, which were four patients with compound heterozygous variants in the *PRKN* and one case with a homozygous mutation of the *PINK1* gene. The novel mutations might reduce the stability of the PRKN and PINK1 protein molecules. The frequency of homozygous mutant genotype p.A340T of the *PINK1* in the EOPD cohort was higher than in control (*p* = 0.0001, OR = 5.704), suggesting this variant might be a risk factor for EOPD. To the best of our knowledge, this is the first study of *PRKN* and *PINK1* genes conducted on Vietnamese EOPD patients. These results might contribute to the genetic screening of EOPD in Vietnam.

## Graphical abstract

To identify variants associated with Vietnamese EOPD, we performed sequence analysis of coding region of *PARKIN* and *PINK1* in 112 patients and 112 healthy individuals. Three rare and three novel mutations in total of 14 variants of *PARKIN* and *PINK1* were detected. The novel mutations might reduce the stability of the PRKN and PINK1 protein molecules.

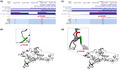

## 1 INTRODUCTION

Parkinson's disease (PD) is a neurodegenerative disorder, with symptoms that are being developed slowly over the years, including moto symptoms (bradykinesia, rigidity, tremor, and postural instability), as well as non‐moto symptoms like cognitive changes, hallucination and delusion, depression, sleep disorders, behavioral changes, constipation, and sensory abnormalities (Paisan‐Ruiz et al., 2004). The proportion of people with PD in the world is about 0.3%, while this rate in people of over 60 years old age is more than 1% (Simon, Tanner, & Brundin, 2020; Tysnes & Storstein, 2017). Early‐onset Parkinson's disease (EOPD) refers to the sickness of patients who have been diagnosed or had onset of motor symptoms before age 50, accounting for 4% of people with Parkinson's disease (Marras et al., 2018).

Genetic, environmental factors, or a combination of both play an important role in the development and progression of PD (Simon et al., 2020). Previous studies have shown that mutations on some genes (*SNCA*, *UCHL1*, *GIGYF2*, *GBA*, *LRRK2*, *PRKN*, *PINK1*, *ATP13A2*, *PLA2G6*, and *FBXO7*) could be the cause of PD, reviewed by Selvaraj and Piramanayagam (2019). Among these, *PRKN* and *PINK1* genes, both involved in a metabolic pathway, are known to be associated with EOPD. *PRKN* is described as the most common cause of autosomal recessive Parkinson's disease (Ferreira & Massano, 2017), accounting for about 49% of familiar EOPD, and 20% of sporadic EOPD (Deng et al., 2006). Meanwhile, more than one hundred mutations have been reported on the *PINK1* gene in families with Parkinson's disease so far (Gelmetti et al., 2017).

The *PRKN* (NM_004562.3, OMIM# 602544) gene, located in chromosome 6, contains 12 exons encoding an enzyme with 465 amino acid residues (Asakawa et al., 2001). PRKN belonging to the group of E3 Ubiquitin ligase (Valente et al., 2001) and plays a vital role in the cell's quality control system with the help of Ubiquitin proteasome system. Mutations in the *PRKN* gene may impair the function of E3 ubiquitin ligase, thereby accumulating proteins that are neurotoxic, especially in the substantia nigra. Until recently, more than two hundreds of mutations have been identified, included nonsense mutation, missense mutation, deletion or insertion of exons in the *PRKN* gene that related to PD (Kitada et al., 1998; Oczkowska, Kozubski, Lianeri, & Dorszewska, 2013; Youn et al., 2019).

The phosphatase and tensin homolog (PTEN)‐induced kinase 1 (*PINK1*, NM_032409.3, OMIM# 608309) located on chromosome 1 (PARK 6 locus), contains eight exons, encodes a serine/threonine protein kinase that localizes in mitochondria with function to protect cells from stress‐induced mitochondrial dysfunction (Valente et al., 2004). Previous studies have found that about 1%–7% of Caucasian EOPD patients have mutations on the *PINK1* gene (Hatano et al., 2004; Valente et al., 2004), while in Japanese patients, it was about 9% (Li et al., 2005).

Although the effects of mutations of the *PRKN* and *PINK1* genes on patients with Parkinson's disease have been extensively studied, mutations of these genes in Vietnamese PD have not yet been investigated. In this study, the coding sequences with exon‐intron boundaries of *PRKN* and *PINK1* genes in 112 Vietnamese EOPD patients and those of the same number of healthy individuals were analyzed.

## 2 SUBJECTS AND METHODS

### 2.1 Subjects

A total of 112 EOPD patients and 112 unrelated healthy controls were subject to our work. The patients were diagnosed based on the UK Parkinson's Disease Society Brain Bank Diagnostic Criteria (Hughes, Daniel, Kilford, & Lees, 1992). All subjects were recruited from the Department of Neurology, 103 Military Hospital, Vietnam Military Medical University, National Geriatric Hospital, and Department of Neurology, University Medical Center Ho Chi Minh City, University of Medicine and Pharmacy at Ho Chi Minh City. Patients with other neurological diseases or history of any other major disease (such as diabetes, hypertension, cardiovascular) were excluded. Control individuals were healthy, without neurological disorders and negative family history of PD. Informed consent was written by all patients and healthy controls who provided blood samples. This study was approved by ethics committees of the Institute of Genome Research, Vietnam Academy of Science and Technology.

### 2.2 Methods

#### 2.2.1 Mutation analysis

Genomic DNA was extracted from the peripheral blood samples using Exgene™ Blood SV (GeneAll Biotechnology, Seoul, South Korea) following the manufacturer's protocol.

Specific primers (available on request) were designed to amplify all coding sequence with flanking region and exon‐intron boundary of *PARKIN* and *PINK1* genes, provided by Phu Sa Biochem Company (Can Tho, Vietnam). PCR reaction was performed with a total volume of 20 µl with 10 ng of total genomic DNA, 0.8 µl of each primer (10 pmole), 1X Neb Master mix (New England BioLabs, Ipswich, Massachusetts, USA), and deionized water. The thermoCycle was 95°C for 5 min, followed by 40 cycles of 95°C for 30 s, 58°C for 30 s, 68°C for 20 s, and a final extension at 68°C for 5 min. The PCR products were purified by Multiscreen PCR 96 Filter Plate (Merck‐Millipore, Burlington, Massachusetts, USA), and sequenced in both directions using ABI Prism BigDye Terminator Cycle Sequencing Kit Version 3.1 (Applied BioSystems, Waltham, Massachusetts, USA), on an ABI 3500 Genetic Analyzer (Applied Biosystems, Waltham, Massachusetts, USA).

Raw sequence data were manipulated by Sequencing Analysis Software (Applied Biosystems, Waltham, Massachusetts, USA) and BioEdit software. Sequence assembly and alignment was implemented by SeqScape 3.0 (Applied Biosystems, Waltham, Massachusetts, USA).

Multiplex ligation‐dependent probe amplification (MLPA) kit (Parkinson probe set P051, MRC‐Holland, Amsterdam, the Netherlands) was used to screen all EOPD patients that carried mutations in *PRKN* and *PINK1* genes, according to the manufacturer's protocol. Exon rearrangement data were analyzed by using Coffalyser.Net software (MRC‐Holland, Amsterdam, the Netherlands).

For control individuals, we sequenced all exons of the *PRKN* and *PINK1* genes that showed to contain variants in the patient cohort.

#### 2.2.2 Statistical analysis

All statistical analyses in this study were implemented using R software. The difference of clinical characteristics between EOPD and control group was calculated with unpaired student *t* test using “ggpubr” package, while the “HardyWeinberg” was used to test Hardy–Weinberg equilibrium. The Fisher extract test was used to analyze genotype distribution between the EOPD and healthy control. Odds ratio (OR) with 95% confidence interval (CI) was implemented with “epitools.”

#### 2.2.3 Variant annotation and prediction

In the present study, in silico prediction analyses were performed to identify the pathogenicity of genetic variants. The effect of variants was predicted by using SIFT (Sim et al., 2012), PolyPhen‐2 (Adzhubei et al., 2010), and MutationTaster (Schwarz, Cooper, Schuelke, & Seelow, 2014), following the default criteria supported by the programs. We used HOPE server (https://www3.cmbi.umcn.nl/hope) (Venselaar, Te Beek, Kuipers, Hekkelman, & Vriend, 2010) to determine the effects of the mutations on amino acid residue property and the impacts on 3D structure of the proteins. Frequent data of variants of global populations as well as mutations associated with Parkinson's disease were extracted from 1000 genomes database (Genomes Project Consortium et al., 2015), gnomAD (Lek et al., 2016), dbSNP, Human Gene Mutation Database (HGMD) (Stenson et al., 2017), and PDgene (Nalls et al., 2014).

The SNV did not present in the 1000 genomes database, gnomAD, and dbSNP, as well as did not report in the literature, was considered as novel SNV.

## 3 RESULTS

### 3.1 Clinical characteristics of EOPD patients

Subjects of the present study were 112 EOPD patients and 112 unrelated healthy individuals. The mean age of patients was 44.5 ± 8.3 years (range from 24 to 74 years old), and the mean age of control group was similar (44.6 ± 4.9), while the mean age at onset of patients was 37 ± 5.91 years (with range 24–49 years). The sex ratio for EOPD and control was the same, with male:female ratio was 54:58. We found that the average stage of Hoehn and Yahr was 2.24, and the mean score of the Unified Parkinson's Disease Rating Scale (UPDRS) part III was 38.28. Thirty‐seven of 112 patients, accounting for 33.03%, showed dystonia. For non‐motor symptoms, there were 38 (33.9%) patients expressed constipation, 51 (45.53%) patients with urinary dysfunction, 47 (41.96%) patients with hyperhidrosis, and 11 (9.82%) patients reported hallucination.

### 3.2 Genetic findings

#### 3.2.1 Common variants in EOPD

In the *PARKIN* gene, coding sequence in 12 exons with intron‐exon boundaries were amplified and directed sequenced in both directions. A total of eight single nucleotide variants (SNV) were found in the patients, including five common, two rare, and one novel variant (Tables 1 and 2). All of five non‐synonymous SNVs were in the Hardy–Weinberg equilibrium, while three intron changes were not fit with.

**Table 1:** The common genetic variants in the *PRKN* and *PINK1* genes

rsID	Variant	Genotype (%)			HWE (*p* value)	Allele frequency		Global Population frequency			In silico prediction effect		
								1000 g	GnomAD		SIFT	PolyPhen‐2	MutationTaster
						Wild	Variant		ALL	EAS			
**PRKN** (NM_004562.3)													
rs1801474	c.500G>A (p.S167N)	GG	GA	AA				0.117	0.067	0.399	T	B	P
	Patient	47	55	10	0.278	0.6652	0.334						
	Control	58	44	10	0.691	0.714	0.285						
	Fisher *p* value	0.228	0.178	NA									
rs1801582	c.1138G>C (p.V380L)	GG	GC	CC				0.172	0.164	0.074	T	B	P
	Patient	98	13	1	0.451	0.933	0.067						
	Control	94	18	0	0.355	0.919	0.080						
	Fisher *p* value	0.567	0.439	NA									
rs3765475	c.872‐68C>G	CC	CG	GG							NA	NA	NA
	Patient	22	31	59	**<0.001**	0.334	0.665						
	Control	6	57	49	**0.04**	0.308	0.692						
	Fisher *p* value	**0.002**	**0.003**	0.246									
rs4709583	c.408‐15T>C	TT	TC	CC				0.950	0.933	0.99	NA	NA	NA
	Patient	1	1	110	**<0.001**	0.0134	0.986						
	Control	0	0	112		0	1						
	Fisher *p* value	NA	NA	NA									
rs3765474	c.872‐35G>A	GG	GA	AA				0.578	0.553	0.70	NA	NA	NA
	Patient	16	35	61	**0.007**	0.2991	0.701						
	Control	6	58	48	**0.02**	0.3125	0.687						
	Fisher *p* value	**0.041**	**0.003**	0.108									
**PINK1** (NM_032409.3)													
rs200708848	c.804A>G (p.L268L)	AA	GA	GG							NA	NA	NA
	Patient	111	0	1	**0**	0.991	0.009						
	Control	111	1	0	0.962	0.996	0.004						
	Fisher *p* value	1	1	1									
rs3738136	c.1018G>A (p.A340T)	GG	GA	AA				0.122	0.092	0.273	T	B	P
	Patient	52	29	31	**0.009**	0.594	0.406						
	Control	62	43	7	0.9	0.746	0.254						
	Fisher *p* value	0.229	0.065	**0.0001**									
rs1043424	c.1562A>C (p.N521T)	AA	AC	CC				0.300	0.291	0.352	T	B	P
	Patient	51	48	13	0.74	0.670	0.330						
	Control	51	50	11	0.804	0.679	0.321						
	Fisher *p* value	1	0.892	0.829									

Note

EAS, East Asian; NA, Not Available; D, Damaging (SIFT, MutationTaster), Deleterious (PolyPhen‐2); P, Polymorphism (MutationTaster); T, Tolerant; B, Benign.

The bold values show the statistical significance ≤ 0.05.

**Table 2:** Rare and novel variants in the PRKN and *PINK1* genes

rsID	Variant	Genotype			HWE (*p* value)	Allele frequency		Global Population frequency			In silico prediction effect		
								1000 g	GnomAD		SIFT	PolyPhen‐2	MutationTaster
						Wild	Variant		ALL	EAS			
**PRKN** (NM_004562.3)													
rs562362828	c.1223A>G (p.K408R)	AA	AG	GG					3.97E‐6	5.43E‐5	T	B	P
	Patient	111	1	0	0.962	0.9955	0.0045						
	Control	111	1	0	0.962	0.9955	0.0045						
novel	c.1240A>G (p.T414A)	AA	AG	GG				.	.	.	D	D	D
	Patient	111	1	0	0.962	0.995	0.0045						
	Control	112	0	0	NA	1	0						
rs778305273	c.1321T>C (p.C441R)	TT	TC	CC				.	5.12E‐5	7.08E‐4	D	D	D
	Patient	109	3	0	0.962	0.986	0.014						
	Control	112	0	0	NA	1	0						
**PINK1** (NM_032409.3)													
novel	c.503C>T (p.A168V)	CC	CT	TT				.	.	.	D	D	D
	Patient	111	1	0	0.962	0.996	0.004						
	Control	112	0	0		1.000	—						
Novel	c.880G>A (p.D294N)	GG	GA	AA							T	D	
	Patient	111	1	0	0.962	0.996	0.004						
	Control	112	0	0		1.000	0.000						
rs35813094	c.1023G>A (p.M341I)	GG	GA	AA				0.0002	0.0002	0.0027	T	B	D
	Patient	111	0	1	0	0.991	0.009						
	Control	111	1	0	0.962	0.996	0.004						

Note

EAS: East Asian; D: Damaging (SIFT, MutationTaster), Deleterious (PolyPhen‐2); P: Polymorphism (MutationTaster); T: Tolerant; B: Benign.

The bold values show the statistical significance ≤ 0.05.

In five frequent changes, p.S167N, p.V380L, c.408‐15T>C, c.872‐68C>G, and c.872‐35G>A, found in our study the frequency of mutant and wild‐type allele was similar in the cohort and control samples, respectively. Similarly, in two common missense substitutions, p.S167N and p.V380L, genotype frequency between EOPD and healthy group was not significantly different (*p* > 0.05) (Table 1).

**Figure 1:**
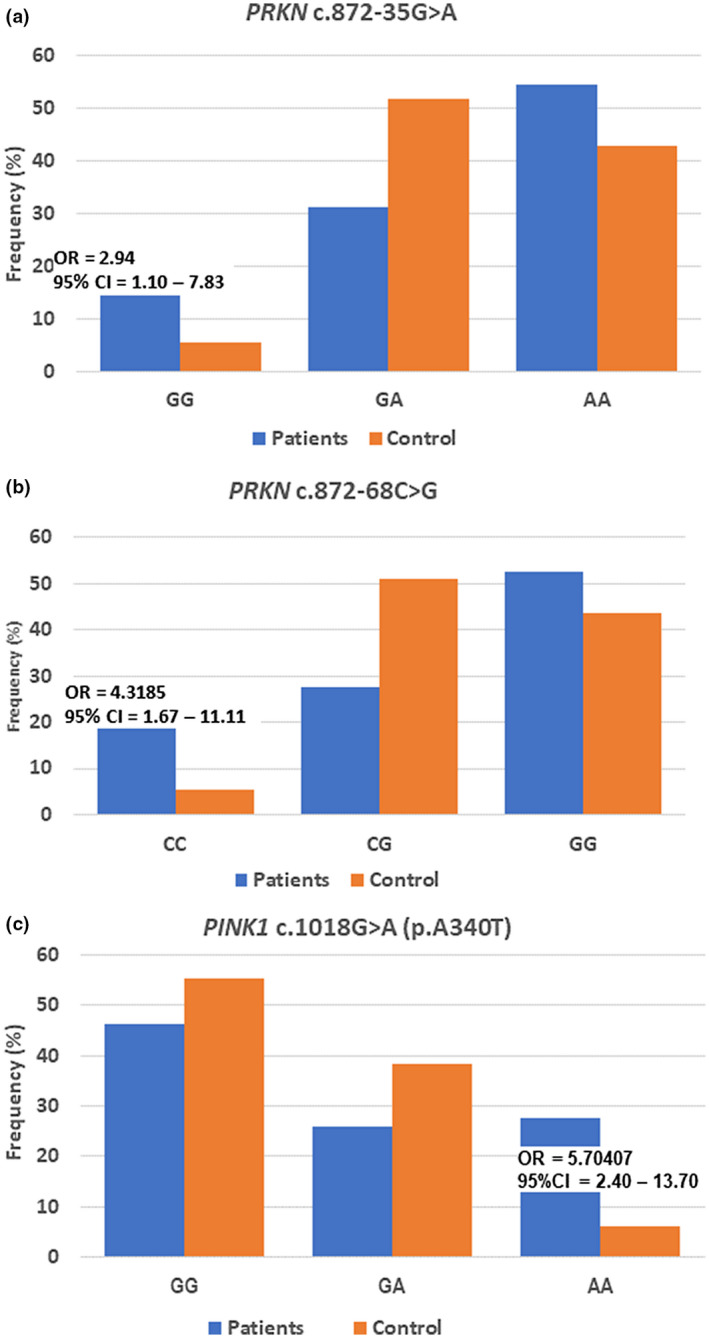
Genotype frequency distribution of variants in the *PRKN* and *PINK1* genes. Frequency distribution of *PRKN* NM_004562.3:c.872‐35G>A (a) and NM_004562.3:c.872‐68C>G (b); (c) Genotype frequency of the *PINK1* NM_032409.3:c.1018G>A in patient and control groups

For three intron variants, the frequency of mutant genotype in EOPD and control group was significantly higher than that in heterozygous and wild‐type genotypes, which was consistent with the data published in the 1000 genome project database (Genomes Project Consortium et al., 2015) and gnomAD (Lek et al., 2016). In particular, we found that in both variants c.872‐35G>A and c.872‐68C>G, the wild‐type homozygous genotype frequency was higher in the disease group than in the healthy group, with *p* = 0.041, Odds ratio = 2.944, 95% CI 1.107–7.830 for c.872‐35G>A and *p* = 0.002, Odds ratio = 4.318, 95% CI 1.678–11.115 for c.872‐68C>G (Table 1, Figure 1a,b).

For the *PINK1* gene, in the EOPD cohort, we identified six changes, all of which were located on the coding sequence (Tables 1 and 2). Among these changes, we have detected three common SNVs, one rare variant, and two novel mutations. Two of three common polymorphisms were missense, and the remaining was silent. Allele frequency of wild‐type and mutant of c.804A>G (p.L268L) and c.1562A>C (p.N521 T) was similar between patient and control groups. Interestingly, in the substitution c.1018G>A ⇒ p.A340T, frequency of allele A in PD was 0.406 and in control was 0.254 (*p* = 0.0009), while genotype AA occurred more frequently in the EOPD cohort than that in the control group (*p* = 0.0001, OR = 5.704, 95% CI = 2.405–13.701, Figure 1c, Table 1).

#### 3.2.2 Rare and novel mutations in EOPD

In the *PRKN* gene, we found two rare SNVs, p.C441R and p.K408R, and a novel substitution c.1240A>G (p.T414A) in five EOPD patients (4.46%) (Table 2). Variant p.K408R was identified in one EOPD case and one control subject in a heterozygous state.

**Figure 2:**
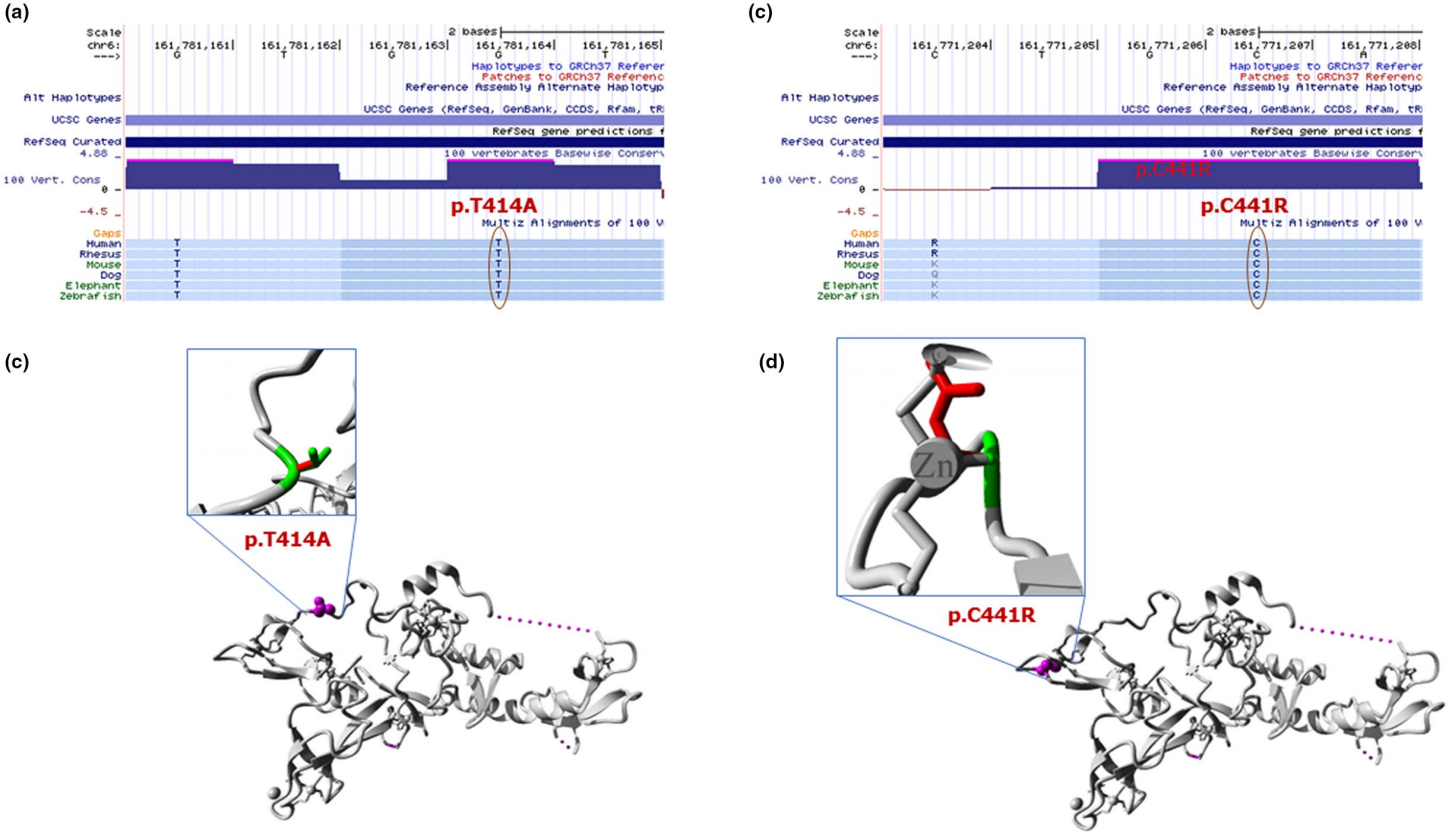
The effect of the mutations of PRKN. UCSC multiz highly conservation of p.T414A (a) and p.C441R (c); Overview of the 3D structure of PRKN molecule (b and d). The protein color was grey, the side chain of the mutated residue was small magenta balls. The side chains of both the wild‐type and the mutant residue are shown and colored green and red, respectively

The novel heterozygous substitution p.T414A of the *PRKN* was detected in an EOPD case but not in the healthy individuals. The result of evaluating the influence on the protein function of this mutation by in silico tools was “pathogenic.” The p.C441R and p.T414A variants were in highly conserved regions across multiple species (Figure 2a,c). The MUpro (Cheng, Randall, & Baldi, 2006) and I‐Mutant2.0 (Capriotti, Fariselli, & Casadio, 2005) tools were used to evaluate the effect of p.C441R and p.T414A variants on molecular stability of PRKN. We found that both C441R and T414A variants might reduce the stability of the PRKN protein molecule.

The heterozygous variant p.C441R presented in three sibling patients of one family but not in the healthy control group and in the 1000 genome database. It presented in the East Asian of the gnomAD database with frequent of 7.08 × 10^−4^ only. The change p.C441R was predicted to have an effect in function of the protein molecular by SIFT (score = 0), PolyPhen‐2 (score = 0.997), and MutationTaster (score = 1) (Table 2). The impact of the variant on the molecular structure of the PRKN was determined by the HOPE server. The result showed that the size differences between the wild‐type and mutant residue could disturb the interaction with the Zn^++^ (Figure 2d). Additionally, with the MLPA method, we also identified hemizygous of exon 3, exon 4, and exon 5 of the *PRKN* in all three patients.

Regarding the *PINK1*, we have identified a rare variant c.1023G>A (p.M341I) only in one patient. The mutant allele occurred in the 1000 genome database and gnomAD with the same frequency of 0.0002, while homozygous mutant genotype AA did not present in the public databases and in our healthy control group as well (Table 2).

**Figure 3:**
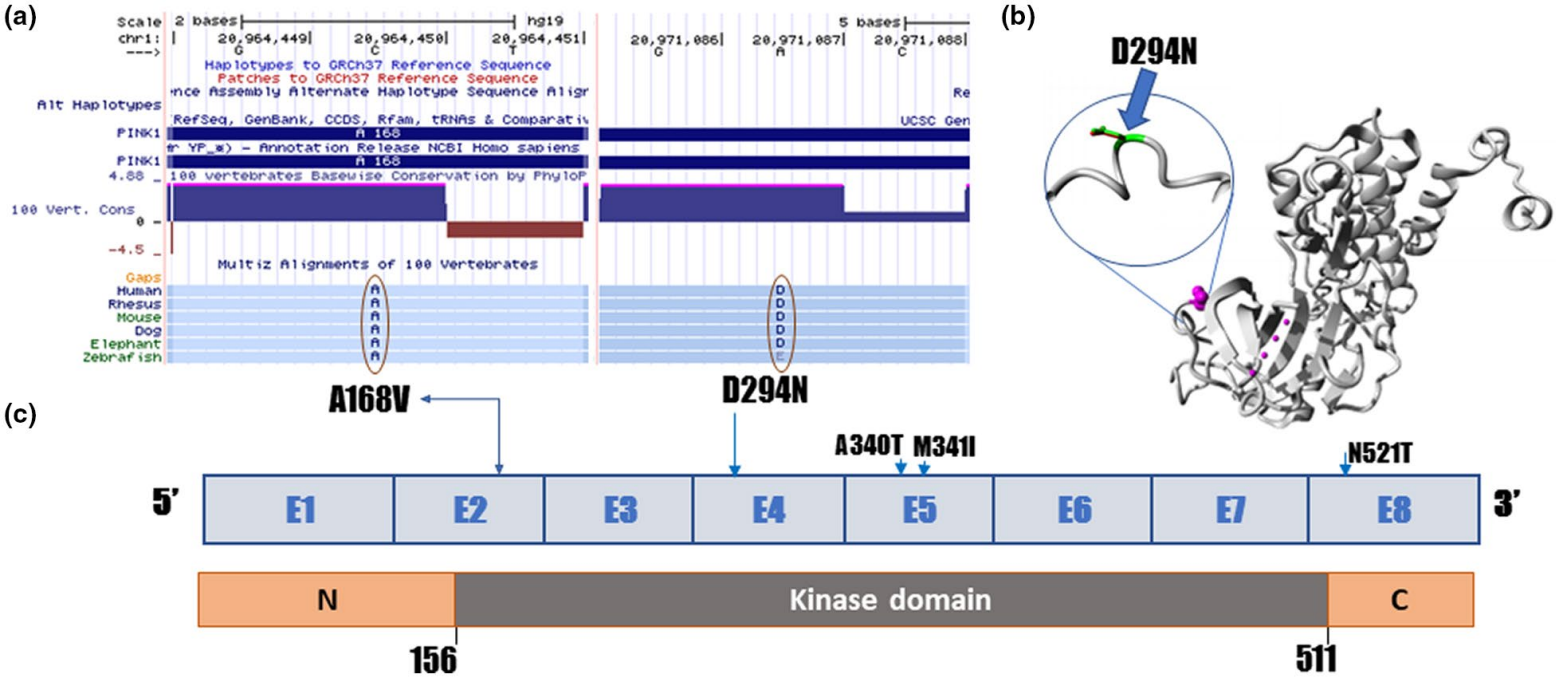
The effect of novel mutations of PINK1. (a) UCSC multiz highly conservation of p.A168Vand p.D294N; (b) Overview structure of the PINK1 protein. The protein color was grey, the side chain of the mutated residue was small magenta balls. The side chains of both the wild‐type and the mutant residue are shown and colored green and red, respectively; (c) Overview structure of *PINK1* gene

Two novel mutations, c.503C>T (p.A168V) and c.880G>A (p.D294N), were identified each in one sample (Table 2). Both mutations were highly conserved among different species (Figure 3a). We used MUpro (Cheng et al., 2006) and I‐Mutant2.0 (Capriotti et al., 2005) tools to evaluate the effect of mutations on the molecular stability of PINK1. The results showed that both mutations decreased the stability of the PINK1 protein. Using the HOPE server, we also determined the impact of these mutations on the molecular structure of the PINK1. Both variations were in the kinase domain (Figure 3b,c). The wild‐type amino acid residue at position 294 was negatively charged, where the mutant form was neutral (Figure 3b). Based on the 3D structure (PDB id: 6EQI), HOPE also showed that the wild‐type residue forms hydrogen bonds and salt bridges with Lysine at position 164. The D294N mutation might reduce or lose interaction with other molecules due to the change in the charge.

## 4 DISCUSSION

In this study, a total of 14 variants were identified in both *PRKN* and *PINK1* of the EOPD patients, including eight common variants, three rare variants, and three novel variants. There was no difference in the frequency of both p.S167N and p.V380L in the *PRKN* gene between analyzed groups, which was consistent with other studies (Gaweda‐Walerych et al., 2012; Martinez et al., 2010; Oliveira et al., 2003; Wang et al., 1999). For variant p.N521T in *PINK1*, we also did not find the difference between two groups, similar to other studies (Chung et al., 2011; Do et al., 2011; Wang et al., 2016).

For the two intron polymorphisms (c.872‐35G>A and c.872‐68C>G) of the *PRKN* gene, although homozygous wild‐type genotype was higher in patients than in healthy subjects, these variants were located on the intron. Therefore, to evaluate the impact of these variants on Parkinson's disease, it is necessary to conduct further studies on molecular mechanisms.

A heterozygous mutation p.C441R of *PRKN* was found on three sibling patients of one family. Besides, these patients also discovered hemizygous of exon 3, exon 4, and exon 5 in the *PRKN*. The mutation C441R was located on RING2 domain of PRKN protein and was predicted as “pathogenic” by all in silico tools. The heterozygous mutation p.C441R combined with hemizygous of exon 3, exon 4, and exon 5 to form a compound heterozygote pattern that mays cause autosomal recessive EOPD.

One patient carried a compound heterozygous mutation in the *PRKN*, which combined a novel putative pathogenic heterozygous mutation c.1240A>G (p.T414A) and a common variant c.1138G>C (p.V380L). The onset of the patient was 24 years old with symptoms of rigidity, postural instability, tremor, and mild dystonia in hands. He had responded well to L‐dopa.

Homozygous mutant genotype (AA) of c.1018G>A (p.A340T) in *PINK1* occurred more frequently in the EOPD cohort than in the control group (*p* = 0.0001, OR = 5.704), and this variant located on kinase domain of PINK1 (Figure 3c). Although this variant has been evaluated as “benign”by in silico prediction tools, we supposed that this variant might be associated with EOPD, similar to the findings of Wang, Feng, Ma, Zou, and Chan (2006) in a study of later‐onset PD of Chinese. Similarly, homozygous mutant genotype AA of the c.1023A>G (p.M341I) might be related to EOPD of the carrier, consistent with another reported (Lee et al., 2009).

One patient carried the p.D294N mutation on the *PINK1* gene and had the p.K408R variation in the heterozygous form on the *PRKN* gene. So far, digenic mutation on the *PRKN* and *PINK1* genes has been found in one patient (Funayama et al., 2008). Based on reports that Parkin and PINK1 (Clark et al., 2006; Park et al., 2006) share a common pathway, the author suggested that digenic mutation of *PRKN* and *PINK1* may affect PD patients. Furthermore, there are hypotheses that a single heterozygous mutation on the *PRKN* or *PINK1* gene may be related to Parkinson's disease (Abou‐Sleiman et al., 2006; Djarmati et al., 2006; Toft et al., 2007).

In this study, we conducted a genetic analysis of *PRKN* and *PINK1* genes in 112 EOPD subjects and 112 controls and found mutations associated with EOPD. Due to the mutation rate of these two genes in EOPD was low, it would be of interest to conduct further investigation on a larger sample size for better statistical analysis of mutations, especially novel and rare mutations.

## 5 CONCLUSIONS

We found mutations of *PRKN* and *PINK1* genes in five (4.4%) patients of EOPD cohort. Compound heterozygous mutations in the *PRKN* were found in four patients, and putative pathogenic homozygous mutation of the *PINK1* was identified in the other one. We also determined the frequency of homozygous mutant genotype p.340T of the *PINK1* in EOPD cohort higher than in the control group (*p* = 0.0001, OR = 5.704), suggesting this variant might be a risk factor for EOPD. Further research with larger sample sizes may be needed for functional analysis of the mutations at in vivo and in vivo to determine their effect on Parkinson's disease.

## CONFLICT OF INTEREST

The authors declare no conflict of interest.

## AUTHORS CONTRIBUTIONS

NDT and NVH: Conceptualization, Methodology, Funding acquisition; NDT, NDS, HTD, NTH, NDB, LTKD, NVB, TNT, NTTH, NHN, MTTH, TTBN, NTD, NHH, and VPN: Resources, Investigation, formal analysis; NDT, NVH: Writing ‐ Original Draft, Writing ‐ Review & Editing.
